# Agreement of rebound and applanation tonometry intraocular pressure measurements during atmospheric pressure change

**DOI:** 10.1371/journal.pone.0259143

**Published:** 2021-10-28

**Authors:** Alice Verticchio Vercellin, Alon Harris, Brent Siesky, Ryan Zukerman, Lucia Tanga, Carmela Carnevale, Fabio Scarinci, Francesco Oddone

**Affiliations:** 1 Department of Ophthalmology, Icahn School of Medicine at Mount Sinai, New York, New York, United States of America; 2 Department of Ophthalmology, University of Miami Miller School of Medicine, Miami, Florida, United States of America; 3 Istituto di Ricovero e Cura a Carattere Scientifico (IRCCS) Fondazione G.B. Bietti per lo Studio e la Ricerca in Oftalmologia ONLUS, Rome, Italy; University of Warmia, POLAND

## Abstract

This study investigated the agreement of intraocular pressure measurements using rebound tonometry and applanation tonometry in response to atmospheric changes in a hyperbaric chamber. Twelve eyes of 12 healthy subjects were included in this prospective, comparative, single-masked study. Intraocular pressure measurements were performed by rebound tonometry followed by applanation tonometry in a multiplace hyperbaric chamber at 1 Bar, followed by 2, 3 and 4 Bar during compression and again at 3 and 2 Bar during decompression. Mean differences between rebound and applanation intraocular pressure measurements were 1.6, 1.7, and 2.1 mmHg at 2, 3, and 4 Bar respectively during compression and 2.6 and 2.2 mmHg at 3 and 2 Bar during decompression. Lower limits of agreement ranged from -3.7 to -5.9 mmHg and upper limits ranged from -0.3 to 1.9 mmHg. Multivariate analysis showed that the differences between rebound and applanation intraocular pressure measurements were independent of atmospheric pressure changes (p = 0.79). Intraocular pressure measured by rebound tonometry shows a systematic difference compared to intraocular measured by applanation tonometry, but this difference is not influenced by changes of atmospheric pressure up to 4 Bar in a hyperbaric chamber. Agreement in magnitude of change between devices suggests rebound tonometry is viable for assessing intraocular pressure during atmospheric changes. Future studies should be designed in consideration of expected differences in IOP values provided by the two devices.

## Introduction

The effects of hyperbaric conditions on human physiology have been the object of many investigations in the last century, including studies of recreational or professional diving and of therapeutic hyperbaric chambers [1]. Of the many physiological parameters investigated, intraocular pressure (IOP) is among the least studied in response to changes of atmospheric pressure (ATM), largely due to the technical difficulties of obtaining accurate and repeatable measurements of IOP using applanation tonometry (AP).

AP is considered the gold standard for IOP measurement in clinical settings and infers IOP from the force required to flatten a predetermined area of central cornea. Two forms of AP are utilized in clinical practice: Goldmann applanation tonometry (GAT), which is considered the gold standard and is attached to a slit lamp, and Perkins AP, a handheld device that allows for more environmental flexibility. The accuracy of AP has been the object of many investigations which have shown AP to be dependent on many different factors including corneal thickness, curvature, rigidity, and axial length [2, 3]. Additionally, since AP is a user-dependent contact procedure, AP requires experienced examiners as well as the use of topical anesthetics and fluorescein, so it may also be affected human error and different sources of artefacts. Meanwhile, repeated AP IOP measurement in a short period of time may lead to induced tonographic artefact secondary to corneal indentation.

Still, despite these challenges, AP has been used extensively in experimental settings because of its reliability. Recently it has also been employed in the study of IOP behavior in hyperbaric conditions induced in hyperbaric chambers [4]. In this particular setting, however, several of the limitations of AP previously highlighted are critical, such as the need to repeat measurements over a short period of time, to repeatedly use topical anesthetics and fluorescein, the risk of corneal abrasions and, ultimately, the risk of tonographic artefacts due to repeated measurement.

Recently, new technologies have been developed in order to obtain accurate, rapid and reliable measurement of IOP while trying to overcome the common limitations of AP. Among these new technologies, rebound tonometry (RB), also known as impact or dynamic tonometry, has shown particular promise as a consistent tool to measure IOP while utilizing a significantly different mechanism than AP [5–7].

The reliability of RB in healthy humans in the clinical setting has also been the subject of several investigations [8, 9]. Generally, RB has shown good agreement with AP, though a consistent difference has been identified in RB measurements, resulting in higher IOP values ranging from 0.5 to 3 mmHg [10–14]. Considering that RB shows good agreement with AP and is a portable and user-friendly alternative to AP that does not require local anesthesia, corneal indentation, or applanation, it may be considered to be a useful tool to overcome limitations associated with measuring IOP in experimental settings involving hyperbaric chambers. Currently, however, there is no information available about the consistency of RB and its agreement with AP in hyperbaric settings. Therefore, this study aims to investigate the use of RB to measure IOP in a hyperbaric chamber and to assess the agreement between RB and AP measurements in response to ATM changes.

## Materials and methods

This prospective, comparative, single-masked study was performed in accordance with the Declaration of Helsinki, and the protocol was approved by the Institutional Review Board of the Fondazione G.B.Bietti, Rome, Italy. A cohort of twelve eyes of twelve healthy subjects were enrolled in this study. Informed consent was signed by all enrolled subjects after all procedures and possible risks were fully explained. All participants were required to meet the following inclusion criteria: age 18 years or older and absence of any active or past ophthalmological disease. Patients were excluded for the following reasons: cardiovascular or respiratory diseases, active or past ear diseases, any psychiatric disease, any use of ocular topical medication, and any history of ophthalmic surgery including refractive surgery.

Pre-examination consisted of standard intake experimentation for hyperbaric treatment, including spirometry, resting and stress electrocardiography, otoscopy, and tympanometry. Ophthalmologic examination included slit-lamp biomicroscopy, undilated fundoscopy, and central corneal thickness (CCT), ocular axial length (AxL), corneal curvature (K) and anterior chamber depth (ACD) measurements.

The experimental procedure involved the use of a multiplace hyperbaric chamber with maximum working pressure of 6 Bar (Galeazzi, Zingonia, Bergamo, Italy) in order to obtain predefined and reproducible increases of ATM. Five consecutive IOP measurements were performed in one randomly selected eye of each subject by RB followed by AP at a baseline of 1 Bar and an ambient temperature of 24°C. ATM was then consecutively increased to pre-set levels of 2, 3, and 4 Bar for consecutive measurements using RB and AP tonometers with a five minute rest period at each ATM level. ATM was then decreased to 3, 2, and 1 Bar with measurements performed again at each level. IOP measurements were performed by the same two trained investigators throughout the experiment (RB: FO, AP: AC). The reader and the measurer were the same person for each tonometer, but each investigator was masked for the IOP values obtained with the other tonometer by the other investigator. The average of 5 consecutive reliable measurements at each ATM level was considered for statistical analysis.

RB was performed using a commercially available rebound tonometer (Icare, Tiolat Oy, Helsinki, Finland). The rebound tonometer is an assembly of two coils coaxial to a probe shaft that bounce a 26.5 mg polybutylene terephthalate magnetized probe off the cornea and detects the deceleration of the probe caused by the eye. A moving magnet within a coil induces changes in the voltage at the two ends of the coil generating a magnetic field with a given voltage, which is then detected by the tonometer sensor. The voltage produced is proportional to the probe speed, which varies according to eye pressure. The probe has several variables linked to movement, but the inverse of its deceleration speed seems to correlate best with IOP [6].

AP was performed using a portable Perkins applanation tonometer (Clement-Clarke International, Harlow, Essex, UK) at each ATM level following RB measurement. A drop of preservative-free topical ossibuprocaine cloridrate 0.4% (Novesina, Novartis Farma S.p.A.) and locally applied fluorescein strips were used before AP but after RB at each ATM level.

During compression, subjects were instructed to perform very light and repeated Valsalva maneuvers to progressively equalize their middle ear pressures to the surrounding pressure. The rate of pressure increase was 0.1 Bar/min from 1 to 2 Bar, 0.05 Bar/min from 2 to 3 Bar, and 0.025 Bar/min from 3 to 4 Bar. The decompression rate was -0.3 Bar/min to allow for easy equalization in all subjects. During compression, due to adiabatic phenomena, the temperature changed to 25, 28.5, 29, 23.5, 20, and 23°C at 2, 3, 4, 3, 2, 1 Bar respectively. Air humidity changed from 74 to 73.5, 70.5 and 72.5% at 1, 2, 3, and 4 Bar respectively, and to 80.5 and 88.5% at 3 and 2 Bar during decompression. Temperature and humidity were not artificially modified during the experiment by cooling or heating systems.

Experiments were performed at the same time of day in three consecutive days and the outside climatologic conditions were sunny, 26°C and 1013 mBar of ATM each day. The hyperbaric chamber was housed in an air-conditioned room temperature of 24°C where all volunteers stayed for 1 hour before starting the experiment.

Descriptive data have been reported as mean ± standard deviation and categorical data have been reported as frequencies. The primary outcome was the agreement between RB-IOP and AP-IOP and was assessed by calculating summary 95% limits of agreement (LoA) at each ATM level from Bland and Altman plots. To test the null hypothesis that ATM variations do not influence the difference between repeated tonometric measurements obtained by AP and RB at each ATM level, a multivariate analysis of variance for repeated measures was performed, including IOP as role variable, ATM level, type of tonometer, and their interaction as factors. Paired t-test was additionally used to test the null hypothesis that AP-IOP and RB-IOP readings at each single ATM level were not different. A p-value < 0.05 was considered statistically significant.

## Results

Twelve eyes of 12 healthy volunteers were included in this study (mean age 43.6±7.2 years, 10 males and 2 females). Mean AxL was 24.1±0.75 mm, mean K was 43.8±0.75 D, mean CCT was 587.4±12.5 μm and mean ACD was 3.74±0.26 mm.

**Fig 1:**
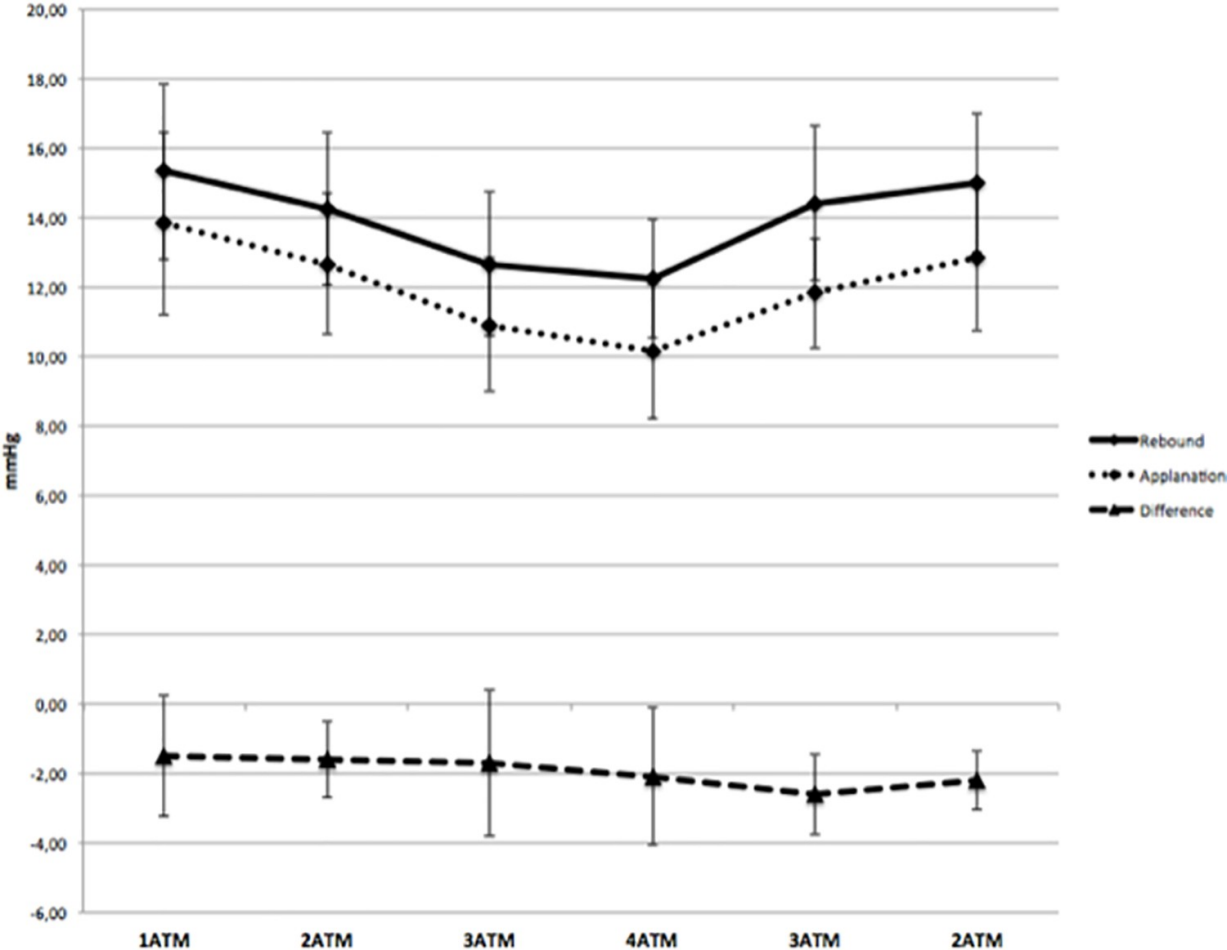
Intraocular pressure measured using applanation tonometry and rebound tonometry at each atmospheric pressure level (ATM) during the experiment in the hyperbaric chamber.

RB-IOP was statistically significantly higher than AP-IOP at each ATM level during both compression and decompression. At 1 Bar before compression, the mean AP-IOP was 13.8±2.6 mmHg and mean RB-IOP was 15±2.5 mmHg (mean difference -1.5 mmHg, LoA -4.9/1.9 mmHg, p = 0.0121). During compression, mean IOP differences between AP and RB readings were -1.6, -1.7 and -2.1 mmHg at 2, 3 and 4 Bar respectively. During decompression, mean IOP differences were -2.6 and -2.2 mmHg at 3 and 2 Bar respectively (Fig 1 and Table 1).

**Table 1:** Mean intraocular pressure (IOP) and its difference with limits of agreement from Bland and Altman plots as measured by rebound tonometry (RB) and applanation tonometry (AP) at each increasing and decreasing atmospheric pressure level during the experiment.

	AP-IOP (mmHg)	RB-IOP (mmHg)	Difference (mmHg)	Limits of agreement (mmHg)	p value
**1 ATM**	13.8±2.6	15.3±2.5	-1.5	-4.9 to 1.9	0.0121
**2 ATM**	12.7±2.0	14.3±2.2	-1.6	-3.7 to 0.5	0.0004
**3 ATM**	10.9±1.9	12.7±2.1	-1.7	-5.8 to 2.3	0.0146
**4 ATM**	10.2±1.9	12.3±1.7	-2.1	-5.9 to 1.8	0.0038
**3 ATM**	11.8±1.6	14.4±2.2	-2.6	-4.9 to -0.3	<0.0001
**2 ATM**	12.8±2.1	15.0±2.0	-2.2	-3.8 to -0.5	<0.0001

Lower limits of agreement between AP-IOP and RB-IOP at each ATM level ranged from -3.7 to -5.9mmHg and upper limits ranged from -0.3 to 1.9 mmHg. Full IOP details and Bland and Altman statistics are reported in Table 1.

Multivariate analysis for repeated measurements, including IOP as role variable, ATM level, type of tonometer, and their interaction as factors, showed that differences between AP-IOP and RB-IOP measurements are not influenced by ATM pressure variations (p = 0.79). Additionally, the model demonstrated that IOP is significantly related to ATM variations (p<0.0001), regardless of the type of tonometer used for measurement.

## Discussion

This study was designed to investigate the use of RB to measure IOP in a hyperbaric chamber, and the agreement between RB and AP measurements during ATM variations. While we observed a difference between RB and AP, the reference standard, we found that this difference does not change with ATM alterations up to 4 Bar, as induced experimentally in the hyperbaric chamber. In fact, this difference was consistent during both compression and decompression, despite different levels of humidity and temperature within the chamber.

Previous research of IOP in hyperbaric chambers is limited to a 2020 study from Albis-Donado et al., which compared IOP measurements from AP and dynamic contour tonometry (DCT) at varying ATM of 1, 1.1, 1.2, and 1.25. They found that DCT was influenced by ATM changes in an inverse relationship to AP [15]. RB, therefore, may potentially present an avenue by which researchers can overcome some of the critical technical difficulties of using AP, and by extension DCT, to measure IOP within hyperbaric chambers. Similarly, since RB is based on the registration of the motion parameters of a rapidly moving probe traveling in the air and rebounding after collision within the eye, it is fair to question whether weather or air density changes (as seen during hyperbaric conditions) may affect the IOP estimation and the reliability of the device in such conditions [4, 5]. Until now, however, the use of RB in these conditions had not been studied.

The results of this study showed that in the presence of wide variations of ATM (up to 4 Bar), the agreement of RB-IOP with the reference standard measurement, AP-IOP, is constant. RB-IOP was found to be consistently higher than AP-IOP at each ATM level, with mean differences ranging from 1.6 mmHg at 2 Bar during compression to 2.6 mmHg at 3 Bar during decompression. This difference is in agreement with previously published studies about the use of RB in healthy humans, where a consistent difference resulting in higher IOP values, as compared with AP, has been described as ranging from 0.5 to 3 mmHg [8–12]. Importantly, however, while the results of this study indicate that measurements obtained by the two tonometers are not interchangeable, the results demonstrate that RB is not affected by ATM and related air density variations induced in the hyperbaric chamber. RB may represent a reliable method to measure IOP during atmospheric changes, but the expected magnitude differences in measurements compared to other applanation techniques, such as the gold standard GAT, should be considered when designing and interpreting studies. Importantly, the Guidelines for the European Glaucoma Society state that there is no consensus for the use of alternative tonometers other than GAT for routine patient care, thus the need to investigate in future studies the use of different tonometers in specific environmental conditions [16].

It is important to note that in this study neither air temperature nor humidity were artificially controlled within the hyperbaric chamber during the experiment. In fact, temperature and humidity were left to variate with the ATM variations (progressively increasing temperature and reducing humidity during compression and vice versa during decompression). This allowed us to conclude that, in addition to air density changes, air temperature and humidity variations do not affect the consistency of RB in relation to AP in this particular setting. Still, while the present study was designed to evaluate agreement between RB-IOP and AP-IOP measurements at different ATM, it also allowed for the observation of the behavior of IOP during compression and decompression up to 4 Bar. According to our data, IOP reduced progressively as ATM was increased from 1 to 4 Bar, with a reverse trend during decompression, regardless of the tonometer used to measure IOP.

To our knowledge, this is the first observation of IOP behavior under varying hyperbaric conditions reaching ATM of 4 Bar. This data is in agreement with data published by Van de Veire et al., where a significant moderate decrease in IOP was observed in association with an increase of ATM up to 2 Bar [4]. Nevertheless, specifically designed studies on larger samples are required to investigate IOP physiology in conditions of increased ATM in healthy subjects. Furthermore, it would be of great interest to explore IOP behavior in response to ATM variations in subjects with aqueous humor dynamic impairments, such as in patients affected by open angle or angle closure glaucoma, who may be at risk of more significant IOP alterations in conditions with altered ATM. Study design could even use topical treatments to alter aqueous humor inflow or outflow. Ultimately, the designs of these future studies could benefit from the results of this study showing that RB is a consistent measurement of IOP within the hyperbaric chamber while taking into consideration expected absolute value differences in IOP compared to other tonometers. When evaluating this study, however, it must be considered that the relatively narrow spectrum of IOP values collected from the sample population was represented by healthy volunteers. Therefore, caution must be used when generalizing these results to populations with higher IOP values, such as patients with glaucoma.
